# Exposure to Criticism Modulates Left but Not Right Amygdala Functional Connectivity in Healthy Adolescents: Individual Influences of Perceived and Self-Criticism

**DOI:** 10.3389/fpsyt.2021.673805

**Published:** 2021-07-06

**Authors:** Sam Luc Bart Bonduelle, Qinyuan Chen, Guo-Rong Wu, Caroline Braet, Rudi De Raedt, Chris Baeken

**Affiliations:** ^1^Department of Child and Adolescent Psychiatry, UZ Brussel/Vrije Universiteit Brussel—VUB (Free University of Brussels), Brussels, Belgium; ^2^Ghent Experimental Psychiatry (GHEP) Lab, Department of Head and Skin, UZ Gent/Universiteit Gent, Ghent, Belgium; ^3^Key Laboratory of Cognition and Personality, Faculty of Psychology, Southwest University, Chongqing, China; ^4^Department of Developmental, Personality and Social Psychology, Universiteit Gent, Ghent, Belgium; ^5^Department of Experimental Clinical and Health Psychology, Universiteit Gent, Ghent, Belgium; ^6^Department of Psychiatry, UZ Brussel/Vrije Universiteit Brussel—VUB (Free University of Brussels), Brussels, Belgium; ^7^Department of Electrical Engineering, Eindhoven University of Technology, Eindhoven, Netherlands

**Keywords:** adolescents, amygdala, functional connectivity, rapid mood changes, self-criticism, perceived criticism

## Abstract

**Background:** Frequent exposure to criticism is a known risk factor for various adult psychiatric disorders. Adolescents may be even more vulnerable to (parental) criticism, as their imbalanced brain maturation makes them prone to stronger mood changes and less effective emotional regulation. Identifying which adolescent subgroups are more vulnerable than others could be of great clinical relevance. Perceived criticism (PC) and self-criticism (SC), two related but distinct traits, could well be crucial vulnerability factors.

**Hypotheses:** After exposure to criticism during fMRI scanning, rapid changes in amygdalar functional connectivity (FC) with other brain areas involved in emotion regulation and social cognitive processing will occur. These changes will depend on trait moderators, such as the adolescents' proneness to (a) perceive others as critical of them (PC) or (b) perceive themselves positively or negatively (SC).

**Methods:** Sixty-four healthy 14–17-year-olds were exposed to a series of auditory comments. Changes in mood states were assessed based on the Profile of Mood States (POMS) prior to and after exposure to these segments. Pre- and post-experiment FC of the left and right amygdalae with other brain areas were also measured. Correlates between FC changes and psychometric measures—including the perceived criticism measure (PCM) and self-perception profile for adolescents (SPPA)—were assessed.

**Results:** First, after being criticized, FC increases of the left amygdala seed region with brain areas related to sustained emotional processing were found, but no right amygdalar FC changes. Second, there was a significant positive partial correlation between individual PCM scores and FC changes between the left amygdala seed region and the left precuneus and left superior parietal cortex, both part of the default mode network.

**Conclusion:** Exposure to criticism resulted in a rapid negative mood change accompanied by an increase in FC between the left amygdala and regions known to be involved in sustained emotional processing, but no right amygdalar FC changes. Furthermore, higher PC but not SC was correlated with stronger left amygdalar FC increases with these regions, suggesting an elevated vulnerability for disturbed emotional processing, as observed in mood disorders, in healthy adolescents with higher PCM scores.

## Introduction

Being criticized is hard. As social beings, we are wired to care about what other people think of us and to pick up on subtle cues of social rejection (1–3). A seemingly mild example of social rejection is criticism, which can actually be quite impactful. Indeed, living in a highly critical environment might well have serious consequences. Repeated exposure to high levels of criticism could lead to increased sensitivity to criticism, resulting in proneness to mental disorders, such as depression (4). It has even been proposed that repeated exposure to severe stressors (such as high levels of criticism or even verbal abuse in more extreme cases) could trigger neurotoxic processes (5), resulting in anatomical and functional brain alterations. For example, in young adults who have suffered severe verbal abuse by their parents as children, gray matter volume alterations have been found in the left auditory cortex (5). Therefore, the well-documented and widely acknowledged long-lasting deleterious consequences of serious early-life stressors do not come as a surprise.

Having highly critical parents may well have an impact on children's self-image. Parental criticism and/or verbal abuse may become internalized over time, resulting in high self-criticism. This is not without importance, as high self-criticism is a major risk factor for several psychiatric disorders, including depression (6–8). Adults who recall their parents as critical, rejecting or overprotecting tend to be more self-critical than those who recall their parents as warm (6, 9–12).

This perception of parental criticism, measured with the Perceived Criticism Measure (PCM) (13), appears to be a good predictor of clinical outcome as well (14). Higher PCM scores have been shown to be consistently linked to greater relapse and poorer symptom course for (adult) patients suffering from depressive, anxiety, obsessive-compulsive and substance abuse disorders (15). PCM scores do not appear to be a proxy for symptom severity, nor are they related to several demographic variables, including gender, educational level and race/ethnicity (15). Whether age influences PCM scores is unclear. The PCM has rarely been assessed in adolescent samples. Thus, it is not known whether the results found in adult samples would hold true in that younger age group, but emerging evidence seems to suggest that it could. In a recent longitudinal study reporting on a large out-patient adolescent sample, higher PCM scores proved predictive of continued clinical depression scores (16). These findings point to the crucial role exposure to and/or perception of criticism may play in (continued) vulnerability for psychopathology.

Adolescents may be especially challenged when it comes to coping with criticism from a neurobiological point of view: their brain areas responsible for emotional reactivity and emotion regulation develop at a different pace, creating an imbalance between the intense emotions that they experience on the one hand, and the underdeveloped coping strategies they are able to apply on the other (17, 18). Teenagers' frontal cortex and frontolimbic connections typically lag behind in maturation (19, 20). This maturation delay has implications for their capacity for emotion regulation, impulse control, foresight and planning (21–23).

By contrast, the limbic system develops earlier, including the amygdala, a key area involved in the rapid detection and processing of various emotional stimuli (24, 25). Hyperactivity of the amygdala is associated with an enhanced response to negative emotional stimuli, as well as being more vulnerable for depression (20). Given the relatively early maturation of the amygdala, adolescents may well be more vulnerable to negative affective stimuli (e.g., criticism) than adults, as their imbalanced brain maturation makes them prone to higher emotional responsiveness and less effective emotion regulation (17). Furthermore, although both the left and right amygdala are part of the limbic system, they may also have distinct functions, even in rapid mood changes. Functional imaging data point to lateralized specialization, with the left amygdala more often activated when processing negative emotional stimuli (26).

Personally salient affective stimuli can have a strong effect on a person's emotions. Several studies have used self-referential auditory segments with a positive (praising), neutral or negative (critical) valence to try and recreate realistic daily-life affective stimuli (and by consequence emotional experiences) in an ecologically valid test situation, including some with adolescents (27–29). Amygdala activity consistently changed after exposure to such auditory segments. Indeed, exposure to parental criticism has been shown to trigger increased activation of neural networks associated with processing negative emotions and decreased activation of cognitive control networks and social cognitive networks in healthy adolescents, suggesting that these neural responses may be normative in minors (28). However, depressed adolescents appear to be even more distraught by negative social evaluation than healthy adolescents, as they display a more pronounced activation of brain areas involved in encoding, retrieving, monitoring and/or evaluating emotionally salient information (29, 30). Self-criticism also appears to activate areas of the brain associated with processing negative information and mentalizing, such as the medial-prefrontal, lateral-prefrontal and anterior cingulate cortex, as well as the insula and amygdala (6, 7, 31).

In adult samples, exposure to criticism has been shown to elicit greater immediate activation of the amygdala in formerly depressed patients than in healthy controls (32, 33). Similarly, greater depressive symptoms were associated with increased activation of the right amygdala in response to criticism in a healthy (but high-risk) adolescent sample (27). Surprisingly, greater depressive symptoms were associated with less activation of the left amygdala after exposure to criticism in this sample.

No previous research in adolescents has studied the impact of perceived criticism (PC) and self-criticism (SC) on the amygdala's functional connectivity (FC), nor on the differences between the left and right amygdalar FC with other brain areas after exposure to criticism. However, this could be very useful as it could help to identify which (healthy) adolescent subgroups are particularly vulnerable for psychopathology (including depression), in order to take preventive measures at an early age and/or provide adequate treatment as early as possible. PC and/or SC could well be crucial vulnerability factors here, which may have neurobiological correlates.

Therefore, in this study, we focus on the changes in FC of the left and right amygdala with other brain areas after exposure to criticism. Overall, we expect FC changes of both the left and right amygdala with other brain areas involved in emotion regulation and social cognitive processing. More specifically, we expect to observe differences based on trait moderators, such as the adolescents' proneness to (a) perceive others as critical of them (perceived criticism) or (b) perceive themselves positively or negatively (self-criticism).

## Materials and Methods

### Recruitment and Clinical Assessment

This study was approved by the UZ Gent Medical Ethics Committee (reference number 2018/0852) and was carried out in accordance with the Declaration of Helsinki (2004). Written informed consent was obtained from participating adolescents and their parents. Participants received a small financial compensation (40 EUR) for taking part in this study, which was part of a larger research project on brain biomarkers for depression.

In total, 96 adolescents participated. They were recruited through a number of schools in the Ghent area, as well as social media pages. Most medical conditions were not a reason for exclusion, unless they formed a risk for magnetic resonance imaging (MRI), such as having metal objects or electronic devices in the body.

For this brain imaging study, we could only include 64 adolescents. Twelve participants were excluded because their dental braces disrupted the scan quality too much. Three participants were excluded because of technical problems with the headphones, which meant the experiment had not been correctly executed. One participant opted to stop the scans before the experiment was performed, citing a headache caused by the scanner's noise as the reason for dropping out. Two more participants were excluded because they interrupted the scans during or just after the experiment, which meant that the post-experiment scans were performed too late. Finally, 14 participants were excluded because of excessive movement throughout the scans. Thus, 32 participants were excluded in total.

Consequently, the study sample consisted of 42 girls and 22 boys between the ages of 14 and 17 (See Table 1). Participants completed the Pubertal Development Scale (PDS), a self-report questionnaire designed to assess development on five indices of pubertal growth. The PDS has demonstrated good reliability and validity (34), and has been used in several studies as an approximate measure of pubertal development (35).

**Table 1:** Demographic variables: participants' gender, pubertal development, and age.

Gender and pubertal development scale (PDS) stage	42 girls (65.6%) Pre menarche: *n =* 1 PDS Stage 3: *n =* 4 PDS Stage 4: *n =* 20 PDS Stage 5: *n =* 17	22 boys (34.3%) PDS Stage 4: *n =* 10 PDS Stage 5: *n =* 12 0 non-binary/other gender
Age	Mean: 16 years, 5 months, 29 days (SD 1 year, 0 months, 13 days) Range: 14 years, 4 months, 13 days−17 years, 10 months, 4 days	

Participants were assessed by a child and adolescent psychiatrist by means of the SCID-5-Junior (36), the Psychotic Disorders section of the K-SADS-PL (37) and the Scale for Suicidal Ideation (38). Participants who met DSM-5 criteria for a depressive disorder, bipolar disorder, psychotic disorder or substance related disorder (current or lifetime) and those at high risk for suicide would have been excluded from the study, as well as participants receiving psychotropic medication, but none had to be excluded for these reasons. Participants would also have been excluded in case of an intellectual disorder; they completed Raven's Standard Progressive Matrices (39) to screen their cognitive ability, but again, none had to be excluded.

Participants completed the Dutch version of the Perceived Criticism Measure (PCM) (13). The PCM is a single-item self-report measure, in which participants are asked how critical significant others (e.g., parents) are of them. In our study, we asked the participants to rate the question “How critical are [your significant others] of you in general?” on a 11-point Likert scale, ranging from 0 (“not critical at all”) to 10 (“very critical indeed”); in other words, higher scores indicated higher perceived criticism. The PCM has demonstrated moderately high test-retest reliability, acceptable convergent validity, and adequate discriminant and predictive validity (40). In our study, we used an adapted Dutch version of the PCM, which has been used in other studies too (41, 42).

Participants also completed the Self-Perception Profile for Adolescents (SPPA) (43). The SPPA is a 35-item self-report questionnaire on six domain-specific self-evaluations, as well as global self-worth. Each item contains two complementary statements, which describe a group of youngsters with either a negative or positive self-evaluation. The respondent is asked which statement describes him or her best, and whether it applies completely or only somewhat to him or her. The items load onto seven subscales: Scholastic Competence, Athletic Competence, Physical Appearance, Social Acceptance, Close Friendship, Behavioral Conduct and Global Self-worth. Global self-worth is considered a non-domain-specific factor, evaluating the respondent's overall feeling about him- or herself. We used the Dutch version of the SPPA, which has been shown to be a reliable and valid instrument with a moderate-to-good fit of the domain-specific six-factor structure (44). For our study, we used the raw total SPPA scores, which have a theoretical range between 35 and 140. Higher SPPA scores indicate a more positive self-perception.

### Medical Imaging and Criticism Paradigm

While in the scanner, participants were exposed to a series of self-referential auditory comments. Each comment was pre-recorded by an adult female voice and lasted 20–30 s, followed by 30 s of silence. The comments were designed to be broadly applicable and relevant to the participants within a family context; unlike some previous studies, they were not personalized. As the original version of this experiment was performed with adults (32, 33), some comments were adapted to better suit an adolescent audience. All adaptations were made in consultation with the original experiment's main author.

Every participant heard the same series of comments through non-ferrous gradient-damping headphones in the following order: two neutral comments, two praising comments, another two neutral comments and finally two critical comments. This specific order was chosen in accordance with the affective contrast theory, which states that the effect of emotional information is larger when it is preceded by the contrasting emotional content (45), in line with previous studies with adult samples (41, 42). Participants were instructed to focus their gaze on a fixation cross, which was projected on a mirror inside the scanner.

Before and after listening to the segments, participants reported on their momentary mood states (MMS), based on the Total Mood Disturbance Score (TMDS) index of the Profile of Mood States (POMS) (46). These MMS were assessed using six statements: participants were asked to rate how tired, vigorous, angry, tense, sad and cheerful they felt at that moment, on a 11-point scale from 0 (“not at all”) to 10 (“very much”). These MMS have also been used in previous studies by our research group evaluating the effect of an experiment on the participants' mood state (42, 47–49).

As stated above, this study is part of a larger research project examining various brain biomarkers of adolescent depression. Other brain scans were made, including functional and structural imaging. Besides the questionnaires described above, other behavioral state and trait questionnaires were completed as well. These data will be further analyzed and published as separate papers.

### MRI Data Acquisition

All neuroimaging data were collected on a 3T Siemens Trio Tim scanner with a 64-channel head coil. For each participant, high-resolution T1-weighted 3D images were obtained using a MP-RAGE sequence with the following parameters: repetition time (TR) = 2,250 ms, echo time (TE) = 4.18 ms, flip angle = 9°, field of view (FOV) = 256 × 256 mm^2^, data matrix = 256 × 256, voxel size = 1 × 1 × 1 mm^3^, 176 slices. Then two resting state (rs) fMRI scans were obtained before and after the criticism paradigm using a single-shot gradient echo planar imaging (EPI) sequence (TR = 2,400 ms, TE = 27 ms, voxel size = 3 × 3 × 3 mm^3^, flip angle = 90°, FOV = 192 × 192 mm^2^, data matrix = 64 × 64, slice thickness = 3 mm without inter-slice gap, 48 axial interleaved slices). During these resting state scans, participants were asked to stay awake with their eyes closed.

### Data Analysis

Rs-fMRI data were pre-processed using SPM12 (http://www.fil.ion.ucl.ac.uk/spm). For each subject, fMRI images were first corrected for acquisition time delay between different slices, and then realigned to the first volume for head motion correction. Subsequently, functional images were co-registered to individual high-resolution structural images and then spatially normalized to the Montreal Neurological Institute (MNI) standard space. The normalized images were spatially smoothed with a Gaussian kernel of 8 mm full-width-at-half-maximum (FWHM). Twenty-four participants were excluded because of wearing braces, and eight subjects were excluded due to the mean framewise displacement (FD) exceeding 0.3. To remove possible spurious variances from the BOLD signal, nuisance signal regression was performed by regressing out (i) six head motion parameters and their temporal derivatives (50); (ii) non-neuronal sources of noise estimated using the anatomical component correction method (aCompCor, the top five principal components from white matter and the top five from cerebrospinal fluid mask); (iii) first-order Legendre polynomial. Finally, residual time series were used to perform amygdala seed-based FC analyses after temporal filtering (band-pass: 0.01–0.1 Hz).

Bilateral amygdala seed regions were selected according to the Automated Anatomical Labeling (AAL) atlas (51). Correlation maps were obtained by computing the Pearson's correlation coefficients between the time courses of the given seed and other voxels in the brain. These maps were converted to z-scores by applying Fisher's r-to-z transformation to improve normality of distribution.

### Statistical Analysis

All behavioral results were analyzed using Statistical Package for the Social Sciences (SPSS) version 26 (IBM, Chicago). The significance level was set at *p* < 0.05, two-tailed, for all analyses. Given that all behavioral data (PCM scores, total SPPA scores, POMS TMDS) were not normally distributed, we applied nonparametric statistics. To investigate the effect of criticism on amygdalar FC, a paired sample *t*-test was used to map the differences of amygdala seed-based FC maps between pre- and post-criticism FC, with age, gender and mean FD as covariates. The significance level was set at *p* < 0.05 cluster-level family-wise error (FWE) correction, with uncorrected voxel-wise *p* < 0.001 by the cluster-forming threshold of 140 voxels. Furthermore, we used Pearson's partial correlation coefficient to assess the relationship between amygdalar FC changes and PCM or total SPPA scores, with age, gender and mean FD as covariates.

## Results

### Psychometric Measures

In our sample, the median PCM score was 6.00 and the median total SPPA score was 107.00 (See Table 2). There was no significant Spearman's rho correlation (*ρ*) between the participants' PCM and total SPPA scores. There were no significant differences between girls and boys, nor between younger and older adolescents; age did not correlate significantly with the PCM or total SPPA scores either.

**Table 2:** Median PCM and total SPPA scores; pre-experiment TMDS and post-experiment TMDS, as well as interquartile ranges (IR) between brackets.

	**Median PCM** ** scores (IR)**	**Median total** ** SPPA scores (IR)**	**Median TMDS** ** pre-experiment** ** (IR)**	**Median TMDS** ** post-experiment** ** (IR)**
Entire study sample (*n =* 64)	6.00 (2.00)	107.00 (15.00)	14.50 (9.00)	21.00 (9.00)
Girls (*n =* 42)	6.00 (2.00)	106.50 (14.00)	13.50 (9.00)	20.00 (10.00)
Boys (*n =* 22)	6.50 (3.00)	108.00 (19.00)	16.50 (8.00)	21.50 (11.00)
14–15 years old (*n =* 20)	6.00 (3.00)	109.50 (12.00)	12.50 (10.00)	21.50 (10.00)
16–17 years old (*n =* 44)	6.00 (2.00)	106.50 (15.00)	16.00 (9.00)	20.50 (10.00)

A Wilcoxon signed-ranks test pre vs. post-criticism showed a significant increase in the participants' total mood disturbance scores (TMDS) after exposure to criticism: *Z* = −5.419, *p* < 0.001. Indeed, the median TMDS went from 14.50 pre-criticism to 21.00 post-criticism (See Table 2). There were no differences between boys and girls regarding pre- and post-criticism TMDS (nor delta TMDS), nor between younger and older adolescents.

There were no significant correlations between the participants' PCM scores and their pre-experiment, post-experiment or delta TMDS. The pre-experiment TMDS showed a significant inverse correlation with the TMDS. The delta TMDS was significantly correlated with the total SPPA score (See Table 3). Of note, partial correlation analyses (controlled for age) did not influence these observations.

**Table 3:** Spearman's rho (*ρ*) correlations between PCM and total SPPA scores, and pre-experiment, post-experiment, and delta POMS TMDS.

	**PCM scores**		**Total SPPA scores**	
	***rho (*ρ*)***	***p***	***rho (*ρ*)***	***p***
POMS Pre-experiment TMDS	−0.015	0.909	–**0.274**	0.039^*^
POMS Post-experiment TMDS	0.080	0.530	−0.166	0.190
POMS Delta TMDS	0.121	0.339	**0.330**	0.008^**^

* *p < 0.05;*

** *p < 0.01. The bold values are statistically significant*.

### Functional Connectivity

**Figure 1:**
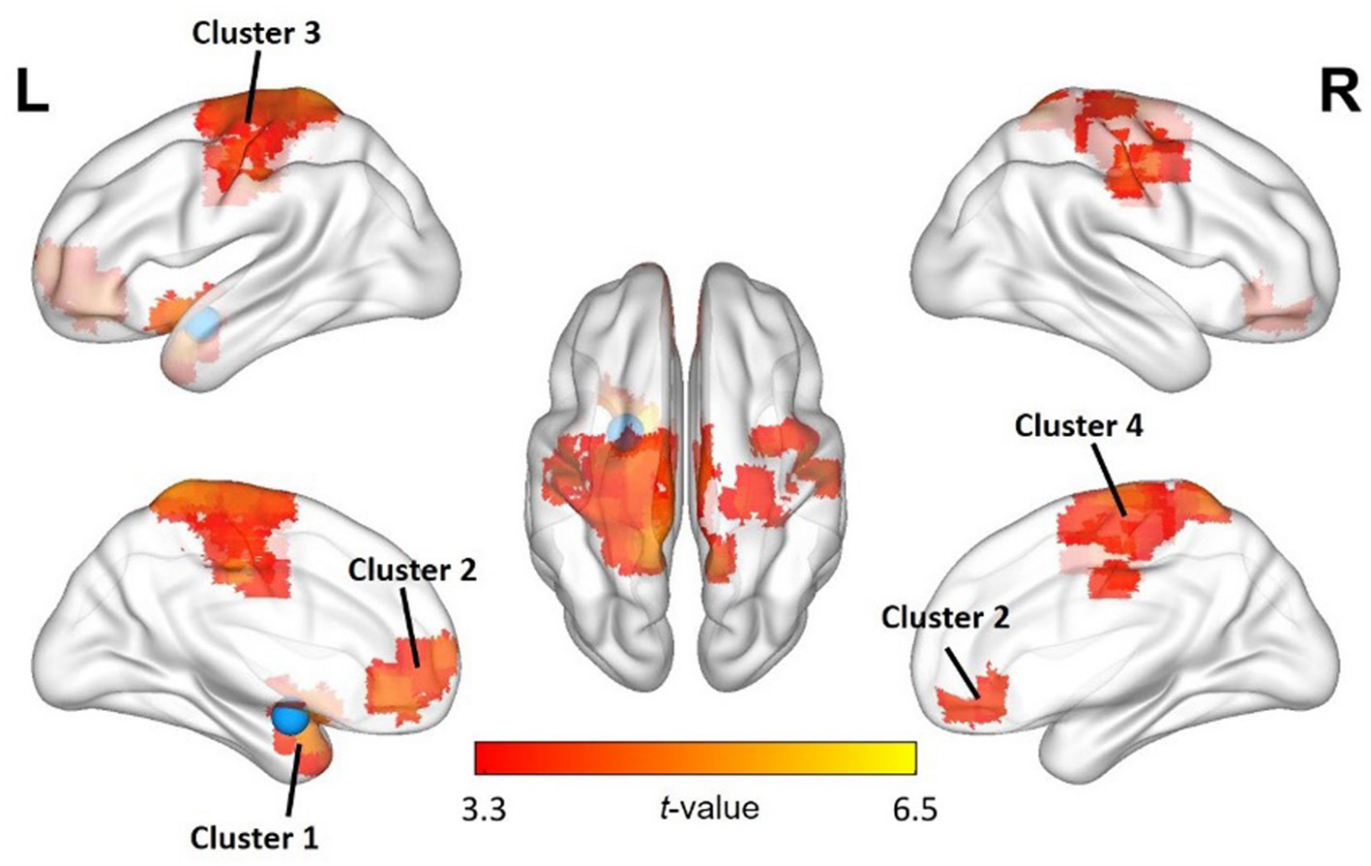
Changes in FC of the left amygdala with other regions after exposure to criticism. A paired sample *t*-test shows significant FC increases between the left amygdala seed region (blue) and several other brain regions. The significance level was set at *p* < 0.05 cluster-level FWE correction, with uncorrected voxel-wise *p* < 0.001 by the cluster-forming threshold. See also Table 4.

First, to assess functional connectivity effects of being criticized, we performed paired sample *t-*tests (pre- and post-criticism). For the left amygdala, these yielded four significant FC clusters, which were located in the left putamen (k = 194; peak MNI coordinates x = −18; y = 6; z = −12), the left mediofrontal orbital cortex (k = 153; peak MNI coordinates x = −3; y = 39; z = −9), the left precuneus (k = 1,121; peak MNI coordinates x = −9; y = −42; z = 72) and the right precentral gyrus (k = 140; peak MNI coordinates x = 51; y = −9; z = 45). No significant FC clusters were found for the right amygdala. There were no differences between boys and girls regarding FC clusters. For a full overview of the implicated regions, see Table 4 and Figure 1.

**Table 4:** Brain clusters with significant differences in FC changes for left amygdala after exposure to criticism.

**Cluster**	**Cluster size** ** (# of voxels)**	**Location**	**MNI coordinates of peak voxel**			***t*-value**
			***x***	***y***	***z***	**(df = 60)**
1	194	Left Putamen	−18	6	−12	6.50
2	153	Left Mediofrontal Orbital Cortex	−3	39	−9	4.55
3	1,121	Left Precuneus	−9	−42	72	4.97
4	140	Right Precentral Gyrus	51	−9	45	4.34

*MNI, Montreal Neurological Institute*.

**Figure 2:**
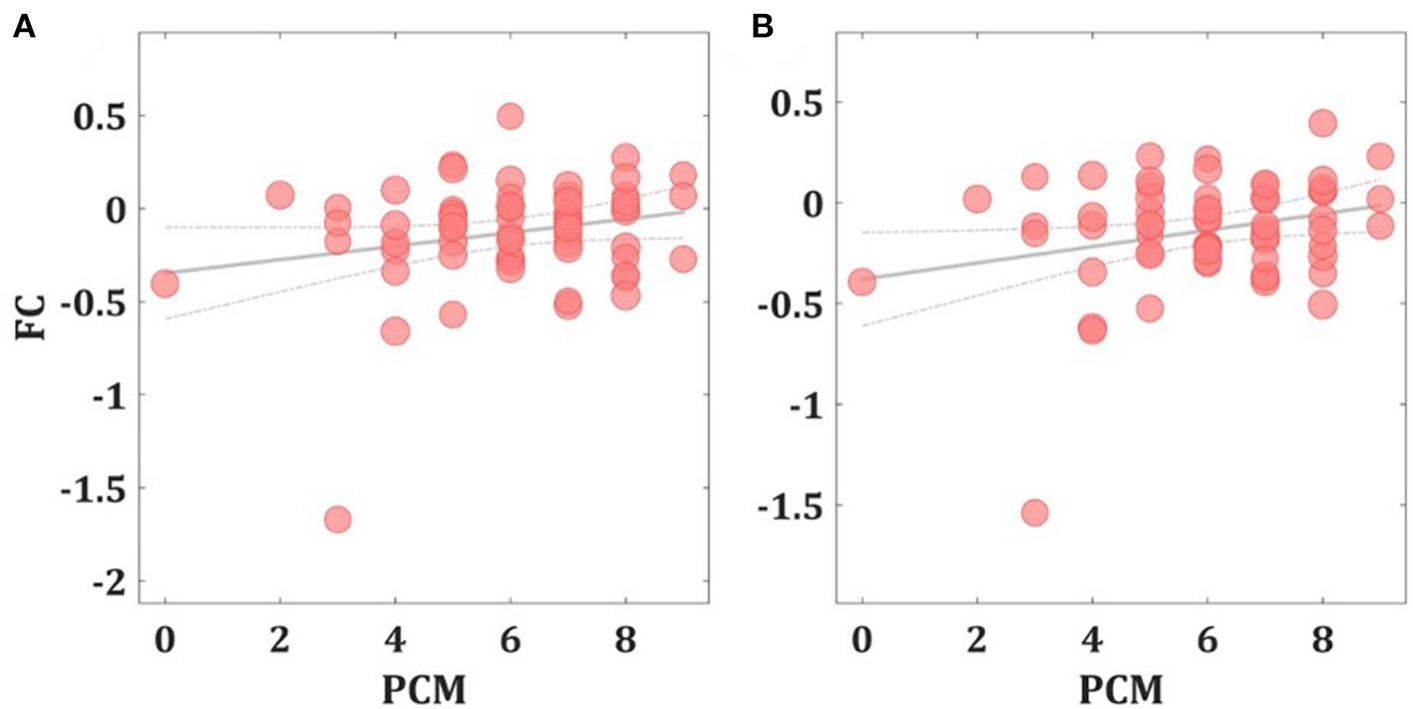
Correlation between FC changes and PCM scores. Scatter plots showing the significant positive Pearson's partial correlation coefficient (*r*) increases between the left amygdala FC seed region and the left precuneus **(A)** and the left superior parietal cortex **(B)**.

Second, to assess the relationship between left amygdala FC changes and the PCM or total SPPA scores, Pearson's partial correlation coefficient analysis showed a significant positive correlation between the individual PCM scores and the FC between the left amygdala seed region and the left superior parietal cortex (*r* = 0.295; *p* = 0.021) and the left precuneus (*r* = 0.322; *p* = 0.011) (See Figure 2). No significant left amygdala FC correlations were observed with total SPPA scores (*p*'s > 0.05).

## Discussion

Our study aimed to examine changes in functional connectivity (FC) of the left and right amygdala with other brain areas after eliciting a negative mood change through exposure to criticism. This is the first study to examine differences between the left and right amygdala FC changes in response to being criticized in adolescents, as well as the first to study the influence of two trait moderators on these FC changes: (a) perceived criticism (PC) and (b) self-criticism (SC).

After exposure to the series of auditory segments (with two critical comments at the end), our healthy adolescent sample experienced a significant increase in mood disturbance, as measured with the POMS TMDS. PC (PCM scores) was not correlated with this rapid mood change. Higher SC (lower total SPPA scores) was correlated with the pre-experiment TMDS, which implies that more self-critical adolescents were in a more negative mood at baseline. Lower SC participants were in a more positive mood at baseline. However, their mood was more susceptible to criticism, as their increase in mood disturbance (delta TMDS) after the experiment was more pronounced. In other words, exposure to criticism had a stronger effect on the less self-critical participants' mood than on the more self-critical ones, who were already in a more negative mood state prior to the experiment.

These findings are largely in line with our expectations. In a similar study conducted with a mixed (healthy, formerly depressed and currently depressed) female adult population, there was an overall increase in negative emotional states after exposure to maternal criticism, but no differences were found between high vs. low PC groups (4). In another study with healthy young adult women, mood disturbance increased significantly following exposure to critical comments (41, 42). Again, there was no significant effect of PCM scores on mood disturbance. In a third study with female adolescents (aged 12–16 years) using a similar study protocol, praise and criticism were assessed separately and led to significant changes in positive and negative emotional states in the expected directions. PC was not measured in this study (27).

For the left amygdala, FC changes were observed with four distinct clusters, located in (i) the left putamen, (ii) the left mediofrontal orbital cortex, (iii) the left precuneus and (iv) the right precentral gyrus, all related to emotion regulation. Acute social stress has been linked to FC changes between the amygdala and the left putamen, part of the dorsal striatum. This brain region is involved in habitual and automatic behavioral responses; its FC with the amygdala has been shown to increase after stress induction, indicating a shift from a flexible and controlled response to more habitual behavior under stress (52). The left mediofrontal orbital cortex is part of the social cognitive network, which helps us understand and interact with other people (53, 54). Stronger FC between the mediofrontal orbital cortex and the amygdala has been linked to more successful downregulation of emotional responses to negative stimuli (55). The left precuneus was at the center of the largest FC cluster. It is part of the default mode network (DMN), a network related to mind wandering and ruminative processes (56). Finally, there was a significant FC change with a cluster around the right precentral gyrus, which houses the primary motor cortex. It is home to one of the clusters reliably activated across studies on emotion regulation (57) and is thus part of the cognitive control network.

For the right amygdala, no significant FC changes were observed after exposure to criticism. The fact that we only found FC clusters with the left amygdala may seem surprising, given that both the left and right amygdala are known to be activated by affective stimuli. However, there appear to be lateralized functional differences as well. In fact, across functional imaging studies, the left amygdala seems to be activated more often than the right amygdala when processing (negative) emotional stimuli. A meta-analysis on this topic came to the conclusion that “the predominant left amygdala activation was not significantly related to stimulus type, task instructions, differential habituation rates of the left and right amygdalae or elaborate processing” (26). A more recent meta-analysis contradicted this finding though, indicating that the lateralized functional differences were due to differences in the temporal dynamics of amygdala activation: emotional stimuli appear to elicit a shorter response in the right amygdala than in the left amygdala (25). Our findings are in line with the latter viewpoint. The resting-state fMRI scans following the criticism paradigm were made ~4 min after exposure to the auditory segments. During this 4-min delay, perfusion scanning (by means of arterial spin labeling—ASL) was performed, which will be discussed in a future paper. Given this study set-up, the faster and shorter neuronal response in the right amygdala may already have been over by the time the post-criticism scan was performed, allowing the right amygdala's FC to return to baseline. The left amygdala on the other hand tends to display a more sustained response and is likely involved in a more elaborate stimulus evaluation (25), which fits well with our findings.

Furthermore, we expected the observed FC changes to be (partially) attributable to trait moderators, such as the adolescents' proneness to (a) perceive others as critical of them (PC) or (b) perceive themselves positively or negatively (SC). The participants' PCM scores showed a statistically significant partial correlation with the increase in FC between the left amygdala seed area and (i) the left superior parietal cortex and (ii) the left precuneus, both part of cluster 3 (See Table 4). The left superior parietal cortex has been shown to be functionally connected with other areas involved in emotion regulation (57) and is part of the DMN (56), just like the left precuneus (58), which has shown increased activation in self-referential conditions (59), and during ruminative thinking (60). This may suggest that adolescents who perceived their closest relationships as more critical of them showed a stronger FC between brain areas linked to processing negative emotions, self-referential thinking and rumination when exposed to criticism. This concurs with the assumption that high PCM scores are a risk factor for poorer clinical course in depressed adults (14, 15) and adolescents (16). Previous research has demonstrated that depressed adolescents display an even more pronounced activation of brain areas involved in encoding, retrieving, monitoring and/or evaluating emotionally salient information after exposure to parental (maternal) criticism than healthy adolescents (29, 30). Our results suggest at least that there is a similarity between the neurobiological response to criticism in healthy adolescents with a high PCM score and adolescents with a depressive disorder. This may substantiate the assumption that higher PCM scores could be a risk factor for the development of a depressive disorder.

There was no statistically significant partial correlation between the participants' SPPA scores and the left or right amygdala FC changes that occurred after exposure to criticism. This finding is not in line with previous studies on neural correlates of self-criticism, which had identified several brain areas, including the amygdala, as linked to this process (6, 7, 31). One explanation could be that in contrast to these studies, our experiment was not set up to induce self-criticism (i.e., “a more self-critical state”), but rather to explore how self-criticism (i.e., a high/low “self-critical trait”) might influence the response to criticism from significant others. In Longe et al. and Kim's et al. studies, participants were presented with statements that described either a negative or a neutral scenario (e.g., “A third job rejection letter in a row arrives in the post”) and were asked to imagine themselves in that scenario being self-critical (and what their self-critical thoughts would be) or self-reassuring (and what their self-reassuring thoughts would be). In other words, they were instructed to be actively self-critical. In our experiment, participants were not given these instructions, but instead exposed to positive, neutral and negative remarks voiced by a motherly figure, designed to be broadly applicable and relevant to adolescents within a family context. Thus, they were processing external criticism, rather than their own criticism, which may well have activated different brain regions.

### Strengths and Limitations

Compared to previous studies on adolescents' neurobiological response to criticism, our study was conducted with a relatively large sample (*n* = 64) of healthy adolescents within a specific age range (14–17 years), which is a major study advantage. The participants' mental health was assessed extensively: each participant was evaluated by a child and adolescent psychiatrist by means of standardized interviews, questionnaires and an intelligence test.

Nevertheless, some limitations need to be mentioned as well. Instead of personalized segments recorded by their own mothers, the adolescents in our study were all exposed to the same sequence of auditory segments. Being criticized by an unfamiliar “motherly” voice rather than their own mothers and hearing generally relevant critical remarks rather than personalized criticism, may well have diminished our participants' emotional response. On the other hand, thanks to the use of a standardized series of auditory segments, any variation between our participants was less likely due to the specific content or tone of the segments they heard. It would have been interesting to have the same series of comments pre-recorded by an adult male voice, to mimic paternal (though unfamiliar) criticism. After all, adolescents may respond differently to motherly than to fatherly criticism. In this study, we chose a female voice in line with previous studies with adult and adolescent participants.

Concerning the PCM, rather than a 10-point Likert scale (scores between 1 and 10), we used a 11-point scale (scores between 0 and 10), in line with some previous studies (41, 42). This way, the scale is transformed to more closely match a ratio scale (with an absolute zero point), which is relevant because we use this scale as a continuous variable in our correlation analyses. It is also worth pointing out that the phrasing of our question about the perception of criticism was not directly related to the participants' parent(s) specifically, but to the general perception of criticism by their significant others (including their parents). Therefore, it reflects a general perception of their closest relationships, rather than a specific relationship, e.g., with their mother (as has been the case in some previous studies using the PCM).

Instead of using another instrument specifically designed to assess self-criticism, such as the self-criticism subscale of the Depressive Experiences Questionnaire for Adolescents (DEQ-A) (61), the Forms of Self-Criticizing/Attacking and Self-Reassuring Scale (FSCRS) (62) or the Levels Of Self-Criticism scale (LOSC) (63), we used the SPPA, which measures self-perception, as a proxy for self-criticism.

The time between the final (critical) auditory segment and the post-criticism FC scan was ~4 min. Given the faster emotional processing by the right amygdala, this may have been too long to detect a change in FC between the right amygdala and other brain areas.

## Conclusion

Being criticized results in rapid negative mood changes in healthy adolescents. On the neurobiological level, whereas FC increased between the left amygdala and other brain areas involved in identifying, (re-)appraising, processing and regulating emotional stimuli, no changes were observed regarding the right amygdala's FC. Moreover, higher PCM scores were correlated with increased FC between the left amygdala and areas that are part of the DMN. In other words, adolescents who perceived their closest relationships as more critical of them, showed a stronger functional connection between brain areas linked to processing negative emotions, self-referential thinking and rumination after being criticized. This may suggest an elevated vulnerability for disturbed emotional processing, as observed in mood disorders. Follow-up studies are needed to assess how these neurobiological and psychometric correlates of vulnerability for depression evolve over time and whether they are indeed predictors of depression at a later stage of life. Future studies could also explore differences in response to criticism dependent on who is expressing criticism (e.g., mothers vs. fathers; parents vs. unfamiliar adults).

## Data Availability Statement

The raw data supporting the conclusions of this article will be made available by the authors, without undue reservation.

## Ethics Statement

The studies involving human participants were reviewed and approved by UZ Gent Medical Ethics Committee. Written informed consent to participate in this study was provided by the participants' legal guardian/next of kin.

## Author Contributions

SB, CBr, RD, and CBa participated in the conception and design of this study. SB and CBa were involved in data acquisition. SB, QC, G-RW, and CBa undertook the data preprocessing and statistical analysis. SB, QC, G-RW, CBr, RD, and CBa participated in data interpretation and in writing the manuscript. SB, CBr, RD, and CBa edited and finalized the manuscript. All authors contributed to the article and approved the submitted version.

## Conflict of Interest

The authors declare that the research was conducted in the absence of any commercial or financial relationships that could be construed as a potential conflict of interest.

## Abbreviations

DMN: default mode network
FC: functional connectivity
FD: framewise displacement
FOV: field of view
FWE: family wise error
IR: interquartile range
MMS: momentary mood states
MNI: Montreal Neurological Institute
PC: perceived criticism
PCM: perceived criticism measure
PDS: pubertal development scale
POMS: profile of mood states
Rs-fMRI: resting state functional magnetic resonance imaging
SC: self-criticism
SD: standard deviation
SPPA: self-perception profile for adolescents
TE: echo time
TMDS: total mood disturbance score
TR: repetition time.
