# Supplementary material for: Exposure to Criticism Modulates Left but Not Right Amygdala Functional Connectivity in Healthy Adolescents: Individual Influences of Perceived and Self-Criticism
Source: Front Psychiatry. 2021 Jul 6;12:673805. doi: 10.3389/fpsyt.2021.673805 (PMC8290839; doi:10.3389/fpsyt.2021.673805)
Supplement: Supplementary file 1 [file Data_Sheet_1.docx]

**Positive, negative and neutral auditory comments**

1. **Criticism / negative comments**

1. One of the things that bothers me about you is how bad you are at dealing with negative feedback. If someone says anything even remotely critical of you, you tend to get very defensive. You are far from a perfect person -- even if that is how you like to see yourself. I really wish you would listen when other people tell you what bothers them rather than getting all hostile and trying to defend yourself.

*Eén van de dingen die me storen aan jou, is hoe slecht je bent in het omgaan met negatieve feedback. Wanneer iemand nog maar nauwelijks iets kritisch zegt over jou, heb je de neiging om zeer defensief te worden. Je bent verre van een perfect persoon – ook al is dat hoe je jezelf wil zien. Ik zou echt graag willen dat je luistert naar anderen wanneer ze je vertellen wat hen stoort, eerder dan vijandig te worden en jezelf te proberen verdedigen.*

2. One of the things that bothers me about you is that you sometimes gossip about people behind their backs. I know you don’t mean to be bad but this is something that is really upsetting. When someone tells you something that is private, it isn’t ok to share that with other people and to disclose another person’s personal information. You may think it’s harmless but people would be really upset if they found out.

*Eén van de dingen die me storen aan jou, is dat je soms roddelt over mensen achter hun rug. Ik weet dat je het niet slecht bedoelt, maar dit is iets dat echt verontrustend is. Wanneer iemand je iets vertelt dat privé is, is het niet oké om dat te delen met andere mensen en de persoonlijke informatie van een andere persoon te onthullen. Je kan denken dat dit onschadelijk is, maar mensen zouden echt overstuur zijn als ze erachter zouden komen.*

3. One thing I don’t like about you is how you never deliver on your promises. You will tell people you are going to do something and then it either never happens or you don’t do it when you say you will. It’s not ok to just do things when you feel like it. Sometimes, people are really relying on you. You need to follow up on things, even if it’s inconvenient for you.

*Iets dat ik niet leuk vind aan jou, is dat je nooit doet wat je belooft. Je vertelt mensen dat je iets zal doen, en dan gebeurt het ofwel nooit, of je doet het niet wanneer je gezegd had dat je het zou doen. Het is niet oké om dingen enkel te doen wanneer je daar zin in hebt. Soms vertrouwen mensen echt op jou. Je moet de dingen opvolgen, ook als het lastig is voor jou.*

4. One thing that really bothers me about her is that she always has to get her own way. She has a hard time taking no for an answer and she can get really resentful when she doesn’t get what she wants. She doesn’t seem to realize that there needs to be some give and take if you are going to get along with people. She has a lot of trouble with relationships and this is one of the reasons why.

*Iets dat me erg stoort aan jou, is dat je altijd je zin moet krijgen. Je kan er moeilijk mee om wanneer je iets geweigerd wordt, en kan heel rancuneus worden wanneer je niet krijgt wat je wilt. Je lijkt niet te beseffen dat je zowel moet geven als nemen, als je wilt overeenkomen met anderen. Je hebt veel moeite met relaties en dit is één van de redenen hiervoor.*

5. Another thing that bothers me is how lazy and apathetic she can be sometimes. She often tends to just sit around, basically doing nothing but vegetating. And she can watch mindless stuff on TV for hours and hours. She says she’s bored but that’s because she has no life. How can you have a life if you don’t make any effort? She needs to try much harder than she does.

*Iets anders dat me stoort, is hoe lui en lusteloos je soms kan zijn. Je hebt vaak de neiging maar wat rond te hangen, zonder iets te doen. En je kan urenlang naar onzinnige tv-programma’s kijken. Je zegt dat je je verveelt, maar dat komt omdat je niets doet met je leven. Hoe kan je ook iets doen met je leven als je geen enkele moeite doet? Je zou veel meer je best moeten doen.*

6. One thing that really bothers me about her is her unwillingness to take any real responsibility. She never follows through with things and she just expects everyone else to accommodate to her and make things right when she messes up. If you say anything to her about it she’ll just say, “Well, I’m under a lot of stress.” There’s always an excuse!

*Iets dat me erg stoort aan jou, is dat je niet bereid bent om enige verantwoordelijkheid op te nemen. Je zet maar zelden door en verwacht gewoon dat alle andere mensen zich naar jou schikken en de problemen oplossen wanneer jij het verprutst. Als men je erop aanspreekt, zeg je gewoon ‘Wel, ik heb veel stress.’ Je hebt altijd een excuus!*

7. One of the things that bothers me about her is that she is not very considerate of other people and she can be very self-involved at times. She does a lot of things without thinking about how it will affect other people. Sometimes it’s just small things – like being polite, that kind of stuff. Other times she acts as if she just doesn’t care about anyone but herself. It’s all about her and what she needs. Forget about anyone else.

*Eén van de dingen die me aan jou storen, is dat je niet veel rekening houdt met andere mensen en bij momenten erg met jezelf bezig bent. Je doet veel dingen zonder na te denken over de mogelijke gevolgen voor anderen. Soms gaat het over kleine dingen, zoals beleefd zijn bijvoorbeeld. Maar op andere momenten gedraag je je alsof je om niemand geeft behalve jezelf. Het gaat altijd over jou en wat jij nodig hebt. Vergeet de anderen maar.*

8. One of the things that bothers me about you is your tendency to talk so much about yourself. Sometimes you just go on and on about what you are doing, what you want to do, how you feel about things. It’s very rare for you to encourage other people to talk. You probably think that other people are interested in what you have to say. But you need to know that a lot of the time it comes across as self-centered, not to mention boring.

*Eén van de dingen die me storen aan jou, is je neiging om de hele tijd te praten over jezelf. Soms blijf je gewoon doorgaan over wat je doet, wat je wilt doen, hoe je je voelt over dingen. Je moedigt bijna nooit iemand anders aan om te praten. Je denkt waarschijnlijk dat andere mensen geïnteresseerd zijn in wat je te zeggen hebt. Maar je moet weten dat het een groot deel van de tijd egocentrisch overkomt, om niet te zeggen saai.*

1. **Praise / positive comments**

1. One thing I really like about you is the way you pay attention to the people you are with. You really seem to be able to make the people around you feel good. Part of it is that you are a good listener. But you also have a really warm personality and a genuine interest in other people. It’s a great combination and it makes people feel really happy to be around you.

*Iets dat ik echt leuk vind aan jou, is de manier waarop je aandacht besteedt aan de mensen waar je bij bent. Je lijkt echt goed te zijn in mensen rondom jou goed te doen voelen. Dat komt deels omdat je een goede luisteraar bent. Maar ook omdat je een warme persoonlijkheid hebt en een oprechte interesse in andere mensen. Het is een geweldige combinatie en het maakt mensen erg blij om in jouw buurt te zijn.*

2. One thing I really like about you is how committed you are to the things you believe in. You really think about things and you seem to know what is truly important in life. You have a lot of insight and understanding about who you are as a person and I think that other people respect you for that. I really admire your integrity and the values you have as a human being.

*Iets dat ik echt leuk vind aan jou, is hoe toegewijd je bent aan de dingen waar je in gelooft. Je denkt diep na over dingen en je lijkt te weten wat echt belangrijk is in het leven. Je hebt veel inzicht en begrip over wie je bent als persoon en ik denk dat andere mensen je hiervoor respecteren. Ik bewonder je integriteit en de waarden die je hebt als mens.*

3. One of the things I really like about her is her sense of humor. It’s not that she is always telling jokes or anything like that but she can be really, really funny. She has a quirky way of seeing the world that I just love. And when she laughs her whole face lights up and she’s just this adorable person that everyone wants to be with. I just love to see her like that.

*Eén van de dingen die ik echt apprecieer aan jou, is je gevoel voor humor. Het is niet zo dat je steeds grappen maakt of zo, maar je kan wel heel, heel grappig zijn. Je hebt een originele kijk op de wereld, waar ik erg van hou. En wanneer je lacht, dan licht je hele gezicht op en ben je gewoon zo’n aangename persoon waar iedereen bij wil zijn. Ik vind het ontzettend leuk om jou zo te zien.*

4. One of the things I really like about her is how thoughtful she can be. She is really good at reading people’s feelings and understanding what they are going through. She tries very hard to be a good friend and she is so emotionally available to people. She’s just…. she’s a terrific person in that regard.

*Eén van de dingen die ik echt apprecieer aan jou, is hoe attent je kan zijn. Je bent heel goed in het aanvoelen en begrijpen van de gevoelens van anderen en wat ze doormaken. Je doet erg je best om een goede vriend te zijn, en je bent emotioneel heel beschikbaar voor mensen. Je bent gewoon… je bent echt een bijzonder persoon in dat opzicht.*

5. Another thing I really like about her is her determination and strength of character. When things don’t go well for her she really tries hard to work out what’s wrong and then she does all she can to fix it. She is very resilient and strong as a person and this is something I admire in her. She has a lot of inner strength and I am so proud of her for that.

*Nog iets dat ik echt leuk vind aan jou, is je vastberadenheid en sterke karakter. Wanneer de dingen niet goed lopen voor jou, probeer je echt te begrijpen wat er fout zit, en zal je er alles aan doen om het op te lossen. Je bent een heel veerkrachtige en sterke persoon, en dat bewonder ik in jou. Je hebt veel innerlijke kracht en daarom ben ik zo trots op jou.*

6. Something I really like about her is how perceptive she is about other people’s feelings. She has an uncanny ability to read people’s emotions and understand what they are going through. She is very caring and just has a lot of empathy for people. And that’s just something that is really wonderful about her.

*Iets dat ik echt leuk vind aan jou, is hoe opmerkzaam je bent voor de gevoelens van anderen. Je hebt een buitengewoon talent om de emoties van anderen te lezen, en te begrijpen wat ze doormaken. Je bent heel zorgzaam en hebt veel empathie voor mensen. En dat is gewoon iets fantastisch aan jou.*

7. One of the things I really like about you is how loyal you are to your friends. You really care about the people you are close to and you always do what you can to make them feel valued and special. Sometimes you show your caring in small ways. But your friends also know that, if they really need you, you will be there to help them, no matter what. Your loyalty is something that people really value and admire about you.

*Eén van de dingen die ik echt leuk vind aan jou, is hoe loyaal je bent naar je vrienden. Je zorgt echt voor de mensen die dicht bij jou staan en je doet altijd wat je kunt om ze zich gewaardeerd en bijzonder te laten voelen. Soms toon je je zorg in kleine dingen. Maar je vrienden weten ook dat, als ze je echt nodig hebben, je er zal zijn om hen te helpen, ongeacht wat. Jouw loyaliteit is iets dat mensen echt waarderen en bewonderen in jou.*

8. Another thing I really like about you is the way that you think about things. Rather than taking the easy path and just going along with the crowd, you form your own ideas and you have your own opinions. You seem to be able to do this without trying too hard. But it gives you your own unique perspective on the world. I think it reflects how thoughtful and creative you are. It also makes you much more interesting than the average person.

*Nog iets dat ik echt leuk vind aan jou, is de manier waarop je denkt over dingen. In plaats van de gemakkelijke weg te nemen en gewoon mee te lopen met de massa, vorm je je eigen ideeën en heb je je eigen mening. Je lijkt dit te kunnen zonder al te veel moeite. Maar het geeft je je eigen unieke perspectief op de wereld. Ik denk dat het weerspiegelt hoe attent en creatief je bent. Het maakt je ook veel interessanter dan de gemiddelde persoon.*

1. **Neutral comments**

1. One of the things you did today was to go out to lunch. You decided to go out for a sandwich and a drink around noon. You got there before the place got busy so it was pretty easy for you to find an empty table. You were there for about half an hour. You ate your sandwich, drank your water and listened to some music on your smartphone. By the time you left, the place was quite crowded.

*Eén van de dingen die je vandaag gedaan hebt, is gaan lunchen. Je besliste om een broodje en een drankje te gaan halen rond de middag. Je was daar nog vóór het druk werd, dus kon je gemakkelijk een vrije tafel vinden. Je was er ongeveer een half uur. Je at je broodje, dronk je water en luisterde naar muziek op je smartphone. Toen je wegging, was het er behoorlijk druk.*

2. One of the things you did last week was to go out for a walk. It was a clear day. You walked from the house to the nearest corner shop – a distance of about half a mile. When you got there, you looked for a magazine. Then you waited in line to pay for it and also bought a pack of gum at the counter. The whole trip took less than an hour.

*Eén van de dingen die je vorige week gedaan hebt, is een wandeling gaan maken. Het was een klare dag. Je wandelde van je huis naar de krantenwinkel, een afstand van ongeveer een halve kilometer. Toen je daar aangekomen was, zocht je een tijdschrift. Daarna wachtte je in de rij om te betalen en kocht je ook nog een pakje kauwgom aan de kassa. Het hele uitstapje duurde iets minder dan een uur.*

3. One of the things you did today was to go grocery shopping for your mother. You walked to the supermarket to get the things on her grocery list. Your mother wanted to make dinner so you bought some eggs, some cheese, and some tomatoes. You also bought milk, orange juice and some cereal for breakfast. When you got home you brought the groceries to the kitchen and then watched TV.

*Eén van de dingen die je vandaag gedaan hebt, is boodschappen gaan doen voor je moeder. Je stapte naar de supermarkt om de dingen op het boodschappenlijstje te kopen. Je moeder wou avondeten maken, dus kocht je eieren, kaas en tomaten. Je kocht ook melk, fruitsap en ontbijtgranen. Toen je thuiskwam, bracht je de boodschappen naar de keuken en keek je daarna naar tv.*

4. One of the things you did this week was to go to the mall. You wanted to buy a birthday card for a friend of yours. Because it was a week-day afternoon, the mall wasn’t especially crowded, so it didn’t take you very long to buy the card. Then you went into several stores and did some window shopping. You tried on clothes in one store but didn’t wind up buying anything.

*Eén van de dingen die je deze week gedaan hebt, is naar het winkelcentrum gaan. Je wou een verjaardagskaart kopen voor een vriendin. Omdat het een weekdag was, was het er niet zo druk, dus hoefde je niet lang aan te schuiven bij de kassa. Daarna ging je verschillende andere winkels binnen en keek je naar de etalages. Je paste kleren in één winkel maar kocht uiteindelijk niets.*

5. One of the things you did today was to spend some time outside in the fresh air. The weather was quite mild for the time of year. You noticed that there were a lot of clouds in the sky but it didn’t seem as if it was going to rain. Although it was fairly quiet, you were aware of the sound of other people as well as the sound of traffic in the distance.

*Eén van de dingen die je vandaag gedaan hebt, is buiten wat tijd doorbrengen in de frisse lucht. Het weer was vrij zacht voor de tijd van het jaar. Je merkte op dat er veel wolken waren, maar toch leek het er niet op dat het ging regenen. Hoewel het vrij rustig was, was je je bewust van het geluid van andere mensen en van het verkeer in de verte.*

6. One of the things you decided to do today was to clean your room. First you tidied up your closet and cleared the floor of your stuff. Then you put your dirty clothes in the laundry bin and emptied the trash bin in your room. After a short break you also tidied up your desk and took some glasses and plates into the kitchen. By the time you were finished you felt a little tired.

*Eén van de dingen die je vandaag beslist hebt te doen, is je slaapkamer opruimen. Eerst ruimde je je kleerkast op en maakte je de vloer leeg. Daarna deed je je vuile kleren in de wasmand en maakte je de vuilnisbak in je kamer leeg. Na een korte pauze ruimde je ook je bureau op en bracht je enkele glazen en borden naar de keuken. Toen je klaar was, voelde je je een beetje moe.*

7. Another thing you did today was to arrange some of the papers in your schoolbag. A lot of the paper handouts were in there loosely and needed to be filed in the right folder. You also noticed that there were some dogeared scrap paper in there, so you were able to throw those out. The last thing you did was to make a list of the handouts that you needed to copy from one of your friends.

*Iets anders dat je vandaag gedaan hebt, is dat je de papieren in je boekentas hebt geordend. Er zaten veel losse werkblaadjes in, die je in de juiste mappen moest steken. Je merkte ook op dat er een aantal verfrommelde kladblaadjes bij waren, dus deze kon je weggooien. Het laatste dat je gedaan hebt, is een lijstje maken van de werkblaadjes die je nog moest kopiëren van je vrienden.*

8. One thing you did today was to spend some time reading. The weather was not too nice outside so you decided to stay at home and not go out. You found a magazine that you had been planning to read for a while and spent most of the afternoon looking at that. When you had finished, you checked your e-mail, made a short phone call, and then thought about what you wanted to eat for dinner.

*Iets dat je vandaag gedaan hebt, is een tijdje lezen. Het weer viel wat tegen, dus besliste je om thuis te blijven. Je vond een tijdschrift dat je al lang wou lezen en bracht het grootste deel van de namiddag daarmee door. Wanneer je hiermee klaar was, bekeek je je mails, belde je iemand op, en dacht je na over wat je ’s avonds wou eten.*
